# Determining whether coeliac disease case-finding in primary care is better than random testing: a retrospective study

**DOI:** 10.3399/bjgpopen19X101648

**Published:** 2019-06-26

**Authors:** Kim Chandler, Gerry Robins

**Affiliations:** 1 Associate Specialist, Department of Gastroenterology, York Teaching Hospital NHS Foundation Trust, York, UK; 2 Consultant Gastroenterologist, Department of Gastroenterology, York Teaching Hospital NHS Foundation Trust, York, UK

**Keywords:** coeliac disease, primary health care, case-finding, general practice, diagnosis, screening

## Abstract

**Background:**

Over 75% of patients (approximately half a million) with coeliac disease in the UK have not been formally diagnosed.

**Aim:**

To determine if case-finding of coeliac disease is better than random testing in primary care.

**Design & setting:**

A pragmatic study looked at all referrals across a 12-month period (December 2013–November 2014) for coeliac serology testing and the indications for testing across 38 GP practices in a well-defined geographical area in North Yorkshire. There was further follow-up for an additional 12 months to determine conversion of positive serology to duodenal biopsy.

**Method:**

All serology samples sent into York Hospital biochemistry department during the study period were analysed for the indication for testing. Positive results were cross-referenced for duodenal biopsies over the following 12 months on the York Hospital pathology database.

**Results:**

Case-finding of coeliac patients in primary care is no better than random testing of the population. Only 71% of patients with positive serology went on to have a duodenal biopsy in the following 12 months.

**Conclusion:**

More education of the population and of primary care physicians is needed around the indications for checking for coeliac disease. It may be that primary care is not the best place to case-find patients with coeliac disease.

## How this fits in

It is known that only approximately 25% of cases of coeliac disease in the UK have been diagnosed. Opportunistic case-finding by primary care is one of the methods of diagnosing more patients. It is not known how successful this strategy is for checking for coeliac disease. Conclusions from this study are likely to be applicable to other chronic diseases that rely on opportunistic case-finding.

## Introduction

There are a number of diseases that have no, few, or protean symptoms, yet expose patients to potential risks. Owing to constraints such as finite resources, or expensive or invasive tests, it is not possible for all such diseases to be screened for within the general population. In this circumstance, case-finding is recommended;^1^ that is, actively searching for people at risk of the disease from a larger population for a specific purpose, such as flu vaccination for those with chronic obstructive pulmonary disease (COPD). The process of case-finding can be systematic (for example, looking through patient records) or opportunistic (for example, when seeing patients who have associated conditions). With regards to opportunistic case-finding, there are a number of barriers to this strategy being successful. Two significant barriers are the at-risk groups coming into contact with healthcare professionals, and the healthcare professionals being aware that they have an at-risk patient with whom they should discuss testing for the associated disease. Coeliac disease is an example of such a disease in which case-finding is recommended, as large-scale studies suggest a prevalence of approximately 1%.^2^ In coeliac disease, the case-finding is usually an opportunistic process, often occurring in primary care, when patients present with symptoms or other conditions with which coeliac disease is associated. To help healthcare professionals in the UK with this process, the National Institute for Health and Care Excellence (NICE) has produced clinical guidelines for identifying at-risk groups of people with coeliac disease, in whom testing for coeliac disease should be carried out.^3,4^

Coeliac disease is a chronic immune-mediated enteropathy predominantly affecting the small bowel, which is precipitated by exposure to dietary gluten in genetically predisposed people.^5^ Adopting a gluten-free diet still remains the sole intervention for coeliac disease.^6^ However, in the UK only 24% of those who have coeliac disease have been diagnosed,^7^ which means that there are estimated to be nearly half a million people who are unaware that they have coeliac disease; hence, the need for case-finding.

The Yorkshire and The Humber region has the third lowest quoted incidence of coeliac disease (out of seven regions) in England.^7^ To help understand why this might be the case, a retrospective study of coeliac serology requests from a defined geographical area over a 12-month period was undertaken.

## Method

Data were obtained from the York Teaching Hospital biochemistry department over a 12-month period (December 2013–November 2014) for all requests for immunoglobulin A anti-tissue transglutaminase antibodies (anti-tTG), a recognised serological marker for coeliac disease.^2^ Repeat requests and those patients in whom known coeliac disease was given as an indication were excluded from analyses. All positive biopsies from the second part of the duodenum (D2 biopsies) were obtained from the York Hospital pathology database and were cross-referenced to obtain the crude rate of referral for D2 biopsy. York Hospital pathology department serves a population of approximately 500 000.

For the time period that the audit covered, the NICE clinical guideline 86, *coeliac d* *isease:* *recognition and* *assessment of* *coeliac* *disease*, was the most up-to-date guideline available to clinicians.^3^ This guideline, published in May 2009, suggested a number of signs, symptoms, or conditions in which patients should be offered testing for coeliac disease and those which should be considered for testing (Box 1).^3^

Box 1. **List of indications for which patients should be offered, or considered for, serological testing for coeliac disease (NICE clinical guideline 86: *coeliac disease: recognition and assessment of coeliac disease*)**

**Table:** 

Offer testing to children and adults with any of the following signs and symptoms or conditions:	Consider offering testing to children or adults with any of the following:
Chronic or intermittent diarrhoea	Addison's disease
Failure to thrive or faltering growth (in children)	Amenorrhoea
Persistent and unexplained gastrointestinal symptoms, including nausea or vomiting	Aphthous stomatitis (mouth ulcers)
Prolonged fatigue	Autoimmune liver conditions
Recurrent abdominal pain, cramping, or distension	Autoimmune myocarditis
Sudden or unexpected weight loss	Chronic thrombocytopenia purpura
Unexplained iron deficiency anaemia or other unspecified anaemia	Dental enamel defects
Autoimmune thyroid disease	Depression or bipolar disorder
Dermatitis herpetiformis	Down syndrome
Irritable bowel syndrome	Epilepsy
Type 1 diabetes	Low trauma fracture
First-degree relatives with coeliac disease	Lymphoma
	Metabolic bone disease (such as rickets or osteomalacia)
	Microscopic colitis
	Persistent or unexplained constipation
	Persistently raised liver enzymes with unknown cause
	Polyneuropathy
	Recurrent miscarriage
	Reduced bone mineral density
	Sarcoidosis
	Sjögren's syndrome
	Turner's syndrome
	Unexplained alopecia
	Unexplained subfertility

Whether the test was requested from primary or secondary care was recorded, as was the specific primary care centre, or specific ward or outpatient clinic for secondary care requests. The clinical indication for the request was recorded (or ‘no clinical details’ if this was the case). The indications were broadly grouped into 'NICE-approved requests', where the indication was one that NICE stated made patients eligible to be offered or considered for testing, and 'non-NICE approved requests', where the indication fell into neither of these categories.

Categorical data were analysed by means of a χ^2^ contingency table with Yates’ correction. Where a correlation between variables was analysed, Spearman’s Rank correlation test was used. A two-tailed *P* value of <0.05 was considered significant.

## Results

There was a total of 15 183 (male 36.0%) anti-tTG requests: 11 321 from primary care and 3862 from secondary care. Of the 15 183 requests, there were 14 494 unique requests, of which there were 10 984 (75.8%) unique requests from primary care. Of these 10 984 unique requests from primary care, 8584 (78.2%) were NICE-approved. There were 97 requests in children, and 1814 requests for coeliac serology gave no clinical details (12.5% of unique requests). A list of all the clinical indications for checking coeliac serology, as recorded by the York Hospital biochemistry department, is given in Box 2.

Box 2. **List of indications given from primary care for coeliac serology testing**

**Table:** 

NICE-approved indications	Non-NICE approved indications
Tired all the time and/or lethargy	?Cancer, pancreas
Abdominal pain	Prostate symptoms
Nausea and/or vomiting	Vertigo and/or balance problems
Reflux	Fibromyalgia
Bloating	Gender reassignment
Diarrhoea	Gout
Iron deficiency	Hair loss
Anaemia	Headache and/or migraine
Family history	Hypertension
Osteoporosis	Itchy and/or rash
Vitamin D deficiency	Ischaemic heart disease
Osteomalacia	Mental health problem (for example, schizophrenia/depression/insomnia/mania/low mood/neurosis)
Bone pain	Breast cancer
Low impact fractures	Metastatic cancer
Amenorrhoea	Non-specifically ill and/or unwell
Irregular periods	Inflammation
(Sub) fertility	Cognitive impairment
Thyroid disease	Electrolyte disturbance
Type I diabetes mellitus	Haematuria
Primary biliary cirrhosis/primary sclerosing cholangitis	On methotrexate
Autoimmune skin disease	Multiple problems
	Neuralgia
	On nitrofurantoin
	Overdose
	Obese
	On lithium
	On steroids
	Deep vein thrombosis
	Pale
	Dyspareunia
	Palpitations
	Panic attacks
	Pelvic pain
	Vasovagal
	Pneumonia
	Reduced libido
	?Pregnant
	Poor veins
	Bariatric surgery
	Reassurance
	Tonsillitis
	Urinary tract infection
	Pulmonary embolism
	Post-myocardial infarction
	Sore throat
	Sinusitis
	Recurrent infections
	Sore mouth
	Vaginal discharge
	Black toe
	Dementia
	Erectile dysfunction
	On statins
	Shortness of breath

? = suspicion but not certainty.

Of the 10 984 requests from primary care, 124 (1.1%) yielded a positive result compared with 50 (1.3%) of the requests from secondary care (*P* = 0.2). Of the 124 patients from primary care (37.9% male), 89 (71.8%) went on to have a D2 biopsy within 12 months (if a biopsy had not happened within 12 months, it was assumed no biopsy had been undertaken). Of those biopsied, the positive predictive value for coeliac serology testing was 93.6%. Of the five patients who had positive serology but negative biopsies, two had ‘diarrhoea’ as an indication, with ‘abdominal pain’, ‘weight loss’, and ‘rash’ all being the indication for one further patient each (all of these indications bar ‘rash’ would be considered NICE-approved). A total of 95 (76.6%) of the positive requests from primary care were NICE-approved. Of those with positive serology without a previous diagnosis of coeliac disease, the most common indications for serological testing were 'anaemia' (13.0%), 'no details given' (11.3%), 'diarrhoea' (10.5%), and 'abdominal pain' (9.2%). 'Osteoporosis', 'osteomalacia', 'fracture', or similar was given as a clinical indication for 0% of the requests in which a positive serology was found (although 1.7% of all requests from primary care did have indications determined as in the 'bone disease' category by the authors). Percentage of total practice list size tested for coeliac serology during the 12 months varied from 0.04% to 9.39%, with percentage testing positive varying from 0% to 4.17% (see Table 1).

**Table 1.** Percentage of population tested, percentage of requests that were 'NICE-approved', and percentage of requests that were positive over the course of the study period, by individual (anonymised) GP practice

GP	% Centre population tested	% NA requests	% Postive	GP	% Centre population tested	% NA requests	% Positive	GP	% Centre population tested	% NA requests	% Positive
**1**	2.25	73.21	1.79	**15**	3.07	83.33	2.82	**29**	3.13	88.52	1.66
**2**	7.61	74.71	0.00	**16**	3.12	82.47	1.10	**30**	0.78	62.50	0.00
**3**	2.57	67.80	2.94	**17**	0.14	88.10	0.00	**31**	1.88	72.07	0.00
**4**	4.04	74.80	0.32	**18**	0.78	75.14	0.00	**32**	2.16	87.50	1.03
**5**	1.83	60.63	0.00	**19**	8.57	75.00	0.13	**33**	4.28	63.32	0.84
**6**	2.52	79.90	1.57	**20**	1.46	62.50	0.96	**34**	1.57	69.47	0.00
**7**	3.95	79.46	1.01	**21**	1.64	85.71	1.69	**35**	6.68	78.13	1.53
**8**	0.04	47.12	0.00	**22**	1.32	71.52	2.38	**36**	6.92	85.87	1.75
**9**	3.18	80.95	0.00	**23**	0.12	87.89	0.00	**37**	1.04	84.62	4.17
**10**	2.05	89.71	0.00	**24**	2.42	91.67	0.27	**38**	3.43	84.93	1.44
**11**	4.18	75.79	1.59	**25**	3.68	60.16	1.03				
**12**	9.39	71.46	1.74	**26**	3.63	81.41	1.33				
**13**	3.90	78.43	1.16	**27**	3.15	74.09	0.29				
**14**	2.09	59.38	1.52	**28**	3.40	75.94	1.00				

NA = NICE-approved

**Figure 1.**
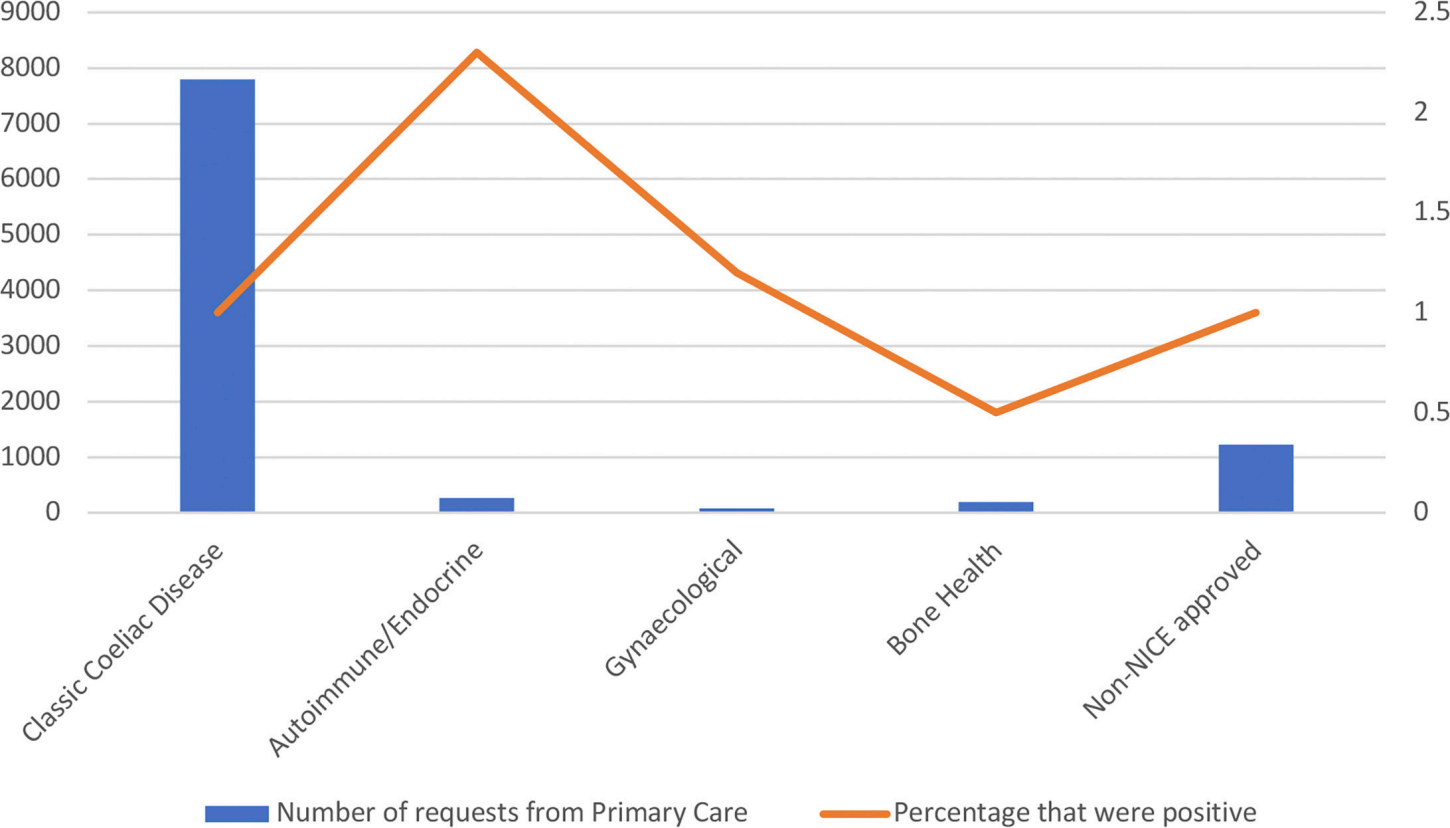
Breakdown of NICE-approved requests and positive serology by indication

A total of 8584 (78.2%) of requests from primary care had a NICE-approved indication for coeliac serology testing and 95 (1.1%) of these requests revealed positive serology. A total of 1216 (11.0%) requests from primary care specified a non-NICE approved indication on the request and 12 (1.0%) of these requests revealed positive serology (which is, in percentage terms, no different to NICE-approved requests). There were 7795 requests from primary care with an indication that was considered to be 'classic coeliac disease' (this was defined by the authors as a gastrointestinal history, including iron deficiency anaemia and family history, and/or lethargy, fatigue, tiredness, and so on); see Figure 1. This group accounted for 73.0% of all requests from primary care and 85 (1.0%) of these requests had positive serology recorded. A total of 262 requests from primary care specified autoimmune and/or endocrine conditions as the indication and six (2.3%) were positive. Eighty-three requests from primary care specified a gynaecological condition on request for coeliac serology. Only 1 (1.2%) was positive. A total of 194 requests from primary care specified a condition related to bone health. Only 1 (0.5%) was positive. A total of 1176 requests had no clinical details (10.7% of GP requests) and 17 of these (1.4%) were positive.

**Figure 2.**
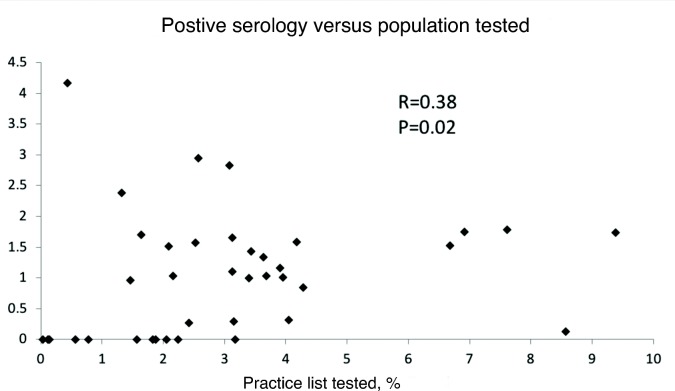
Correlation between percentage of practice population tested and percentage of requests returning positive serology

There was a weak, albeit statistically significant, correlation (*R* = 0.38, *P* = 0.02) between percentage of practice population tested and positive serology (that is, the more patients a practice tests, the greater its pick-up rate; see Figure 2), but there was no correlation between percentage of requests from a practice that were NICE-approved and pick-up rate (*R* = 0.23, *P* = 0.15). Similarly, looking at subcategories of NICE-approved requests (for example, 'classic coeliac disease', autoimmune-related conditions, gynaecological conditions, and bone disease) there was no correlation between percentage of requests within a given NICE-approved subcategory and positive serology (*P*>0.05).

## Discussion

### Summary

The most striking conclusion from this study is that, with an overall positive predictive value of 1.1%, the rate of case-finding in coeliac disease in primary care is no higher than the expected prevalence; that is, it is no better than random testing of patients. This could imply that those patients who are at increased risk of coeliac disease do not attend their GP practice (that is, do not have the opportunity to be assessed as to their risk of coeliac disease). Awareness campaigns, such as that recently undertaken by Coeliac UK, have shown that with advertising, pop-up campaigns, and point-of-care testing, the percentage of people seeing their GP subsequently being diagnosed with coeliac disease is as high as 17%.^8^ An alternative explanation is that the 'wrong' patients are being tested in primary care, which implies an education issue with regards to guidelines, recommendations, and frontline healthcare professionals. With regards the list of indications given, many were outside the NICE guidelines. There are many national recommendations and guidelines that GPs need to be aware of, and trying to stay abreast of all of them is undoubtedly a challenge. Perhaps unexpectedly, those practices that seem to adhere more to NICE-approved indications when requesting coeliac serology fare no better with case-finding than those practices that adhere less well. However, the vast majority of NICE-approved requests from primary care in this study fall into the 'classic coeliac disease' category and it is likely, therefore, that this finding also reflects the need to educate those expected to undertake opportunistic screening that coeliac disease should not just be thought of in its 'classical presentation', but that they should also to consider coeliac disease’s associations with other processes, such as autoimmune disease (including type 1 diabetes), gynaecological conditions, and metabolic bone disease. It is likely that both these factors (that is, at-risk patients not presenting and lack of education and/or awareness among healthcare professionals) are playing a role in the low level of case-finding seen in this study. This also raises the question of how valuable the NICE guidelines actually are with regards trying to identify at-risk groups in primary care (as pick-up rates were no different between NICE-approved and non-NICE approved requests), as well as how the guidelines are interpreted by primary care clinicians. There must also be concern that 29% of patients who tested positive do not seem to have had a D2 biopsy (at least within 12 months of their positive blood test).

The data show a near 200-fold difference in percentage of practice population tested for coeliac disease between the highest and lowest testing practices. It was noted that a number of the lowest testing practices are on the periphery of the geographical area, so this could simply reflect those practices sending more blood requests to other laboratories outside the area. Two of the highest testing practices were close to garrisons within York; therefore, by definition could be expected to have higher turnover rates of patients within any given 12-month period. Further information about drivers of serology testing rates in the different practices would be helpful in this regard. An interesting footnote is that there were 689 duplicate samples taken over the course of the study. This could be argued to be an unnecessary cost to the local health economy (also taking into account the cost of drawing, transporting, and other handling of the blood sample).

### Strengths and limitations

The main limitation of this study is that it relies on what the laboratory has recorded as the indication for undertaking coeliac serology testing. Information recorded by healthcare professionals as indications for testing is often not robust and does not usually give a clear insight into the overall thinking of the healthcare professional requesting the test. For example, the authors would like to know how many patients were tested for coeliac disease simply because a patient requested the test (there has been a significant increase in the last 10 years of people avoiding gluten as a 'lifestyle' choice rather than having coeliac disease, with at least 6% of the American population now describing themselves as 'gluten-sensitive’).^9,10^ Similarly, it would also be interesting to know how many patients on a 'self-imposed' gluten-free diet either declined or were not offered coeliac serology testing as they were not prepared to reintroduce gluten back into their diet (and thus the testing would not be meaningful). Also, data on patients who are in the increased risk categories of developing coeliac disease have not been captured, nor any data on whether: (a) they did not attend their GP practice owing to lack of awareness on their behalf; or (b) did attend their GP practice but were not identified as being at increased risk. The main strengths of this study are that it was undertaken in a relatively small geographical area (approximately 25 miles diameter) in which the vast majority of blood results from primary care come through the study laboratory; the size of the study (over 15 000 samples were analysed); the breakdown of the data by individual GP practice; and follow-up of at least 12 months on all patients. Indeed, in the 12-month period covered by this study, it seems 7% of the York population were tested for coeliac disease.

### Comparison with existing literature

As far as the authors are aware, there are no other studies that have looked at case-finding for coeliac disease in adults in primary care in the UK. There was a very recent study from Sicily that looked at two separate strategies for testing in primary care.^11^ The first strategy involved serial testing of consecutive subjects aged <75 years being offered a point-of-care test (POCT) for coeliac disease. The second strategy involved offering only those with symptoms or associated conditions a POCT, but there was a systematic search for patients with this second strategy. In this study, 1.6% of POCT were positive with the first strategy and 30.5% with the second strategy. Between May 2015–August 2016, Coeliac UK held pop-up events in seven British cities and tested almost 500 people with a POCT test if they had associated symptoms on filling out a simple questionnaire.^8^ A total of 17% of people had a positive test result using the POCT and were referred to their GP for further investigation, but whether these patients did indeed see their GPs and the outcome of this follow-up is not known. A study from Catalonia using point-of-care testing in a smaller area than this study (a population of approximately 100 000) and only 350 patients were consecutively recruited. This study had a pick-up rate of 1.14%.^12^ This was twice previous pick-up rates in this area, although 34/58 of the primary care physicians participating in this study undertook a pre-study questionnaire to assess their knowledge of coeliac disease (the other 24 were invited but declined). Previous studies in primary care in the UK suggest that patients with undiagnosed coeliac disease have more consultations with their GPs in the 5 years prior to diagnosis, implying the opportunity to case-find is there.^13^

### Implications for research and practice

It is recommended that further studies assess whether the findings in this study are repeated in other parts of the UK with regards to coeliac disease (particularly as there have been new NICE guidelines published with regards to coeliac disease^4^ in 2015 and publicity campaigns by Coeliac UK), and with other diseases where case-finding is used as a strategy (for example, COPD and Alzheimer’s disease). This would enable common themes to be seen, from which lessons could be learnt. It may be that as point-of-care testing becomes available for coeliac disease, and if the requirement for at least some adults not to have to undergo biopsy to confirm diagnosis of their coeliac disease (as currently with some children, based on current European Society for Paediatric Gastroentrology Hepatology and Nutrition guidelines),^14^ that case-finding strategies can be more successfully implemented.

## References

[1] NHS England (2015). Using case finding and risk stratification: a key service component for personalised care and support planning. https://www.england.nhs.uk/wp-content/./01/2015-01-20-CFRS-v0.14-FINAL.pdf.

[2] Ludvigsson JF, Bai JC, Biagi F (2014). Diagnosis and management of adult coeliac disease: guidelines from the British Society of Gastroenterology. Gut.

[3] National Institute for Health and Care Excellence (2009). *Coeliac disease recognition and assessment of coeliac disease* [NICE Clinical Guideline No 86].

[4] National Institute for Health and Care Excellence (2015). Coeliac disease: recognition, assessment and management [NICE Clinical Guideline No 20]. https://www.nice.org.uk/guidance/ng20/resources/coeliac-disease-recognition-assessment-and-management-pdf-1837325178565.

[5] Ludvigsson JF, Leffler DA, Bai JC (2013). The Oslo definitions for coeliac disease and related terms. Gut.

[6] Armstrong MJ, Robins GG, Howdle PD (2009). Recent advances in coeliac disease. Curr Opin Gastroenterol.

[7] West J, Fleming KM, Tata LJ (2014). Incidence and prevalence of celiac disease and dermatitis herpetiformis in the UK over two decades: population-based study. Am J Gastroenterol.

[8] Coeliac UK Our campaign successes. https://www.coeliac.org.uk/campaigns-and-research/our-campaign-successes/.

[9] Mansueto P, Seidita A, D'Alcamo A (2014). Non-celiac gluten sensitivity: literature review. J Am Coll Nutr.

[10] Igbinedion SO, Ansari J, Vasikaran A (2017). Non-celiac gluten sensitivity: all wheat attack is not celiac. World J Gastroenterol.

[11] Scoglio R, Trifirò G, Sandullo A (2019). Diagnostic yield of 2 strategies for adult celiac disease identification in primary care. J Clin Gastroenterol.

[12] Esteve M, Rosinach M, Llordés M (2018). Case-finding in primary care for coeliac disease: accuracy and cost-effectiveness of a rapid point-of-care test. United European Gastroenterol J.

[13] Cannings-John R, Butler CC, Prout H (2007). A case-control study of presentations in general practice before diagnosis of coeliac disease. Br J Gen Pract.

[14] Husby S, Koletzko S, Korponay-Szabó IR (2012). European Society for Pediatric Gastroenterology, Hepatology, and Nutrition guidelines for the diagnosis of coeliac disease. J Pediatr Gastroenterol Nutr.

